# Nicotine initiation risk among Polish youth - PolNicoYouth study results

**DOI:** 10.1093/eurpub/ckac131.567

**Published:** 2022-10-25

**Authors:** Ł Balwicki, A Tyrańska-Fobke, D Kaleta, M Goniewicz, K Klimiuk

**Affiliations:** 1 Department of Public Health and Social Medicine, Medical University of Gdańsk, Gdansk, Poland; 2 Department of Hygiene and Epidemiology, Medical University of Lodz, Łódź, Poland; 3 Department of Health Behavior, Roswell Park Comprehensive Cancer Center, Bufflo, USA; 4 Faculty of Medicine, Medical University of Gdańsk, Gdansk, Poland

## Abstract

Smoking and the use of electronic cigarettes pose a risk of cardiovascular disease. The aim of the study was to analyze the initiation of these behaviors among Polish youth. The cross-sectional study was carried out in 2020 on a sample of secondary school students (N = 19241) representative of the Polish population, using the CAWI method. In order to estimate the relationship between the independent variables and the outcome variables, the Bayesian multivariate logistic regression was used in the R program using the brms library. Among Polish youth, age (lnBF = 303.55), smoking of traditional cigarettes by parents (lnBF = 117.29) and the type of school to which youth (lnBF = 36.15) and the province of residence (lnBF = 9.08) attend. Gender, size of the place of residence and parents’ education are not related to the risk of tobacco initiation with traditional cigarettes. When e-cigarettes were tried at least once in their life, a significant correlation was identified with a greater number of factors, such as age (lnBF = 124.87), smoking of traditional cigarettes by parents (lnBF = 56.48), province of residence (lnBF = 23.41), gender (lnBF = 16.53), use of heated tobacco (lnBF = 9.34) and e-cigarettes (lnBF = 9.13) by parents, size of the place of residence (lnBF = 8.61) and school type (lnBF = 7.26). Only the level of parents’ education remains unrelated. Factors related to the initiation and attempted use of nicotine products by adolescents indicate potential targets for the intervention. Parents who smoke and use e-cigarettes and heated tobacco should be made aware that their addiction is a strong risk factor for their children’s health. It can become a motivating factor for quitting the addiction.

**Key messages:**

• Parental health behaviors are factors related to the initiation and attempted use of nicotine products by adolescents and indicate a potential target for a public health intervention.

• Parents who smoke and use tobacco products should be made aware that their addiction is a strong risk factor for their children’s health. It can become a motivating factor for quitting the addiction.

